# Neoadjuvant therapy in non-small cell lung cancer: basis, promise, and challenges

**DOI:** 10.3389/fonc.2023.1286104

**Published:** 2023-12-08

**Authors:** Sukumar Kalvapudi, Yeshwanth Vedire, Sai Yendamuri, Joseph Barbi

**Affiliations:** ^1^ Department of Thoracic Surgery, Roswell Park Comprehensive Cancer Center, Buffalo, NY, United States; ^2^ Jacobs School of Medicine and Biomedical Sciences, State University of New York, Buffalo, NY, United States; ^3^ Department of Immunology, Roswell Park Comprehensive Cancer Center, Buffalo, NY, United States

**Keywords:** neoadjuvant, preoperative, NSCLC, systemic therapy, immunotherapy, chemoimmunotherapy, targeted therapy

## Abstract

**Introduction:**

Survival rates for early-stage non-small cell lung cancer (NSCLC) remain poor despite the decade-long established standard of surgical resection and systemic adjuvant therapy. Realizing this, researchers are exploring novel therapeutic targets and deploying neoadjuvant therapies to predict and improve clinical and pathological outcomes in lung cancer patients. Neoadjuvant therapy is also increasingly being used to downstage disease to allow for resection with a curative intent. In this review, we aim to summarize the current and developing landscape of using neoadjuvant therapy in the management of NSCLC.

**Methods:**

The PubMed.gov and the ClinicalTrials.gov databases were searched on 15 January 2023, to identify published research studies and trials relevant to this review. One hundred and seven published articles and seventeen ongoing clinical trials were selected, and relevant findings and information was reviewed.

**Results & Discussion:**

Neoadjuvant therapy, proven through clinical trials and meta-analyses, exhibits safety and efficacy comparable to or sometimes surpassing adjuvant therapy. By attacking micro-metastases early and reducing tumor burden, it allows for effective downstaging of disease, allowing for curative surgical resection attempts. Research into neoadjuvant therapy has necessitated the development of surrogate endpoints such as major pathologic response (MPR) and pathologic complete response (pCR) allowing for shorter duration clinical trials. Novel chemotherapy, immunotherapy, and targeted therapy agents are being tested at a furious rate, paving the way for a future of personalized systemic therapy in NSCLC. However, challenges remain that prevent further mainstream adoption of preoperative (Neoadjuvant) therapy. These include the risk of delaying curative surgical resection in scenarios of adverse events or treatment resistance. Also, the predictive value of surrogate markers of disease cure still needs robust verification. Finally, the body of published data is still limited compared to adjuvant therapy. Addressing these concerns with more large scale randomized controlled trials is needed.

## 1 Introduction

Lung cancer is the leading cause of cancer related mortality in the United States. As of 2022 21 percent of all cancer deaths can be attributed to lung cancer (1). Non-small cell lung cancer (NSCLC) accounts for the majority (80-85%) of all lung cancer cases (2, 3). Seventy percent of NSCLC patients are diagnosed at an advanced stage, leading to poor survival outcomes (4). With increased use of low dose computed tomography (CT) screening in high-risk individuals, more NSCLC cases are being detected at an earlier stage (5). As the screening protocol for detecting early-stage NSCLC improves, the treatment protocol should evolve as well, to maximize the survival benefit for patients. Early-stage NSCLC is currently treated with curative surgical resection followed by systematic adjuvant therapy. However, long term disease control remains poor, with 25% - 70% of patients having disease recurrence (6–8). The addition of adjuvant platinum-based chemotherapy offers a modest survival benefit of 5% at five years (9).

Recognizing the need to improve survival outcomes in early-stage lung cancer, researchers are increasingly turning to neoadjuvant therapy. Neoadjuvant therapy is defined as systemic anticancer treatment given before surgery (10). When compared to adjuvant therapy, neoadjuvant therapy attacks metastasis earlier; allows for tumor downstaging; increases treatment compliance; allows for researchers to use surrogate endpoints to estimate survival benefit; and permits for a biomarker driven approach to rapidly drive clinical research (10–13). Challenges associated with neoadjuvant therapy in the clinical setting include complication of the surgical field (resulting from post-treatment inflammation and fibrosis) and potential delay to curative resection (14–16).

Several proven therapeutic modalities are providing viable platforms for current and emerging neoadjuvant strategies for treating lung cancer. By far the most studied of these is chemotherapy. The development of neoadjuvant chemotherapy paralleled that of adjuvant chemotherapy in resectable NSCLC from the 1990s up to 2009. Multiple trials have demonstrated comparable outcomes between neoadjuvant and adjuvant therapy. However, due to its simpler implementation as well as earlier availability of survival data from clinical trials, adjuvant chemotherapy was more widely adopted than neoadjuvant chemotherapy.

The emergence of immune checkpoint inhibitors (ICIs) has ushered in a new era of NSCLC treatment possibilities. ICIs block inhibitory signaling that restrain the activity of T-lymphocytes as a means to boost antitumor activity (17). Multiple clinical trials have established the overall survival benefit of monoclonal antibodies targeting the programmed cell death protein 1/programmed death-ligand 1 (PD-1/PD-L1) and the cytotoxic T-Lymphocyte associated protein 4 (CTLA4) in advanced NSCLC (18). Acknowledging the potential of ICIs, neoadjuvant strategies are being employed to gather preliminary safety and efficacy data, efficiently and expeditiously for various ICIs. This approach is proving more rapid and effective than traditional adjuvant trials reliant on survival endpoints (4, 19–22). While the study and development of neoadjuvant immunotherapies are progressing at a relative break-neck pace, some potential stumbling blocks exist with this modality, including resistance to immunotherapeutic agents, particularly when used as monotherapies. Many immunotherapy patients either fail to respond to treatment or develop resistance stemming from several mechanisms. To overcome this challenge and to maximize the antitumor response, numerous studies are investigating potential combinations of neoadjuvant ICI therapy with chemotherapy agents or molecular targeted therapy agents (17, 23, 24). Results of combination therapy trials are promising, but further research has to be done for regulatory approval and optimal benefit (25). Another concern about immunotherapy concerns the occurrence of immune-related adverse events (irAEs). In the neoadjuvant setting, IrAEs can lead to delayed resection and increased morbidity (22, 23). As such, careful monitoring for irAEs and, when possible, measures to prevent them may be essential for successful neoadjuvant immunotherapy in NSCLC patients. Due to the heterogeneity of response to ICI therapy, the development of treatment resistance, and the potential for IrAEs, there exists a need to develop biomarkers to predict response to treatment. So far in the neoadjuvant setting, only PD-L1 status and tumor mutational burden (TMB) have been explored and there is a need for further research to enhance their applicability to the clinical space and develop more biomarkers to maximize clinical benefit (26). Alongside ICI advancements, targeted therapies for specific driver mutations have gained prominence. The era of molecular-based targeted therapy in lung cancer can be said to have started with the identification of activating oncogenic mutations in the epidermal growth factor receptor (EGFR) in NSCLC and subsequent development of the tyrosine kinase inhibitor gefitinib as treatment (27). Evaluation of key driver mutations, such as those found in the genes encoding EGFR, anaplastic lymphoma kinase (ALK), and ROS proto-oncogene 1 (ROS1), has now become routine practice in the diagnostic pipeline of individuals with advanced non-small cell lung cancer (NSCLC). As in neoadjuvant ICI therapy, neoadjuvant targeted therapy in resectable NSCLC is still in its infancy. Some phase II trials have thus far established the feasibility of targeted therapy in the neoadjuvant setting, but many more are underway. Due to the plethora of potential therapeutic molecular targets along with the difficulty of establishing a sizable patient cohort in NSCLC, there has been a recent increase in the popularity of umbrella trials. In such trials, a single disease (in this case NSCLC) is stratified into many subgroups, with each intervention arm being defined by a different molecular target (28). The neoadjuvant setting is ideal for conducting such trials, as it allows for direct evaluation of pretreatment and posttreatment tumor samples (22). Data from phase III trials are eagerly awaited to establish clinical survival benefit of neoadjuvant targeted therapy.

Here we present and discuss a number of recent advances in the development, testing, and performance of these neoadjuvant treatment approaches in hopes of clarifying the current state of the therapy in early-stage lung cancer while highlighting the topics ripe for further development and research.

## 2 Methods

The PubMed medical literature database and search engine of the United States National Library of Medicine at the National Institutes of Health were used on 15 January 2023 to identify published research studies that were possible relevant to this review. The search was limited to English-language articles. A combination of search terms to capture articles reporting on neoadjuvant and adjuvant (“neoadjuvant” or “adjuvant” or “preoperative” or “postoperative”) systemic therapy (“systemic therapy” or “therapy” or “immunotherapy” or “chemoimmunotherapy” or “targeted therapy”) among early-stage NSCLC patients (“early stage” or “resectable” or “local” or “locoregional” and “NSCLC”) were used. A total of 107 articles were considered relevant by two authors following a reading of the abstracts and the full texts were collated for review. We also queried ongoing clinical trials in ClinicalTrials.gov on 15 January 2023 using the search terms “neoadjuvant”, “preoperative”, “chemoimmunotherapy”, “immunotherapy”, “targeted therapy”, “NSCLC stage I” and “NSCLC stage II”, and “resectable NSCLC”. A select number of seventeen ongoing trials were selected and the information obtained for review.

## 3 Results & discussion

### 3.1 Rationale for neoadjuvant therapy

Several motives are driving the exploration of neoadjuvant therapeutic strategies in NSCLC. Most importantly, neoadjuvant therapies are known to combat micro-metastasis during the early phases of disease. Individual cancer cells or small collections of cancer cells shed from the original tumor are generally too few in size and quantity to be reliably detected with current clinical methods (29). However, they are thought to be the primary reason for the development of secondary tumors, cancer recurrence and the underestimation of lung cancer burden, leading to poor survival outcomes (30). Therefore, limiting micro-metastases with neoadjuvant modalities is hypothesized to achieve higher rates of successful margin-negative and/or nodal involvement-negative resection (11).

Another benefit of preoperative systemic therapy is that it can cause clinical nodal downstaging and reduction of primary tumor volume, increasing the potential for surgical resection (12). A 2006 study found that neoadjuvant chemotherapy activity at the primary tumor and mediastinal downstaging were strongly associated with OS in stage III NSCLC (31). Indeed, Pilav et al., found that 30% of stage IIIA NSCLC patients previously deemed inoperable were able to undergo surgery with a curative intent after preoperative chemotherapy (32).

Additionally, multiple retrospective analyses of clinical trials indicate better tolerability of neoadjuvant systemic therapy vs. adjuvant approaches (10). In line with this notion, a 32 study meta-analysis comparing the efficacy of postoperative (n=22) versus preoperative chemotherapy (n=10) found that the percentage of patients who were ultimately able to receive chemotherapy was greater in the neoadjuvant arms when compared with adjuvant group (33). Patients in the adjuvant arms of these studies were less able to initiate chemotherapy due to decreased respiratory performance and postoperative complications. Switching to a neoadjuvant approach in patients at elevated risk for postoperative regimen intolerance could prove crucial in maximizing survival benefit.

The neoadjuvant interval before surgery also allows for a more extensive preoperative workup, permitting the integration of lifestyle modifications into the lung cancer treatment pipeline (10). This could improve the performance status of patients, decreasing postoperative complications and reducing unnecessary hospital stay. For example, even short term smoking cessation was shown to dramatically decrease the frequency of postoperative pulmonary complications in NSCLC patients after curative intent surgery (34). Benefits of pre-surgery exercise interventions have also been reported (35), and while the specific levels and types of activity or dietary modifications best suited for improving post-surgical outcomes remain unclear, it is possible that the lead-time afforded by neoadjuvant treatments may provide for multipronged treatment regimens and synergistic effects.

Finally, the neoadjuvant setting has been characterized as a more robust platform for drug development than the adjuvant setting (13). The traditional drug development process relies on large randomized controlled trials to show the benefits of a specific therapy in terms of OS, a costly and time-taking process. In contrast, the surrogate pathological endpoints used in neoadjuvant clinical trials allow for rapid assessment of treatment efficacy in smaller multi-arm trials. Perhaps this was best exemplified in breast oncology, where the neoadjuvant NeoSphere clinical trial predicted the benefit of pertuzumab five years before the adjuvant trial, APHINITY confirmed it (13, 36, 37). Also, availability of pre- and post-treatment tumor tissue biopsies afforded by the neoadjuvant setting allows for development of biomarker driven approaches. Pre-treatment tissue can also be evaluated for biomarkers predictive of response to treatment while post-treatment tissue can be used to validate those hypotheses and provide critical information about drug resistance or other mechanisms of treatment failure (22). This is particularly valuable in the immunotherapy arena where glimpses of the factors influencing success and failure, unclouded by the influence of other therapies, have been limited.

### 3.2 Assessment of treatment response in neoadjuvant therapy

As briefly mentioned above, the gold standard for evaluating clinical benefit in cancer treatment is OS, but it requires many years to obtain dependable results. One unique feature of neoadjuvant therapy is the ability to study radiological and adaptive pathological responses of the tumor in response to systemic therapy. This can be used to prognosticate treatment response and customize treatment strategies depending on the characteristics of each patient. Response evaluation criteria in solid tumors (RECIST 1.1), major pathologic response (MPR) and complete pathologic response (pCR) are three of the main surrogate endpoints used in place of OS.

The proportion of patients achieving a complete or partial response evaluated by RECIST 1.1 is known as objective response rate (ORR) and is the major radiological surrogate end point in use (38). Here, early change in tumor size is used to stratify patients into the different response categories. RECIST also establishes a standardized language of efficacy for measuring PFS that is easily performed using available radiological equipment and easy to interpret (39). This allows for comparison of the treatment agents across different trials and also lends itself to be easily translated to the clinical space. However, many detractors state that the RECIST poorly predicts OS and PFS due to bias caused by missing data, early dropouts, and differential scheduling of disease progression assessments (40–42). In addition, the correlation between reduction in tumor volume and increase in survival is not established. In a meta-analysis of 14 advanced NSCLC trials, Blumenthal et al. found that there was no significant association between ORR and OS (10). A further complication is that immunotherapy can demonstrate atypical response patterns compared to traditional chemotherapy that RECIST is inadequate in capturing. Patients on immunotherapy can have a delayed but durable response to therapy or demonstrate pseudoprogression, in which infiltration of the tumor by immune cells causes the tumor to appear enlarged on CT (43). Both of these patterns would be falsely labelled as progression using RECIST criteria. In response to this concern, additional scales such as irRC, irRECIST, iRECIST, and imRECIST have been proposed to measure clinical response in ICI therapy (44). Critics argue that these scales only provide marginal benefit while disproportionately increasing complexity of image interpretation and data management (45).

Recognizing the need for improved surrogate endpoints in neoadjuvant therapy, pathologic assessments of response were developed, namely pCR and MPR. Pathologic complete response is defined as the complete absence of residual invasive cancer in resected specimens and all sampled lymph nodes (46). It is the most robust and widely accepted surrogate endpoint in clinical oncology practice. A recent meta-analysis of 28 studies comprising 7011 NSCLC patients found that a median 18% of patients achieved pCR after neoadjuvant therapy. In this study, patients with pCR had significantly better OS than those without (HR = 0.50, 95% CI: 0.45–0.56) (47).

MPR is defined as less than or equal to 10% residual tumor cells in resected lung and lymph node tissue (48). Retrospective analyses by Pataer et al., and Weissferdt et al. revealed that MPR was significantly predictive of long-term OS in neoadjuvant chemotherapy treated NSCLC (49, 50). The potential of MPR as a surrogate for OS gained interest as achieving pCR happens in a low proportion of patients. MPR is achieved in 20-50% of patients, allowing for evaluation of ongoing treatment response in more patients when compared to using pCR exclusively (38).

### 3.3 Disadvantages of neoadjuvant therapy

While the potential benefits of neoadjuvant therapies are numerous, some disadvantages exist as well. Post-treatment inflammation and fibrosis could make minimally invasive resections challenging. Although several authors have reported that VATS or robotic resections are feasible after neoadjuvant immunotherapy, higher rates of conversion to thoracotomy are also seen (51, 52). Neoadjuvant treatment can also lead to delays in surgical resection, the gold standard for treatment of early-stage NSCLC. As such, while sufficient time must be given to allow patients to respond to preoperative therapy, but this period should not overly delay potentially curative surgery (16). Around 10% of patients receiving neoadjuvant chemotherapy or immunotherapy fail to undergo curative intent resection (16). Research into the factors influencing post-treatment adverse events and surgical field changes in patients receiving neoadjuvant therapy is essential to ensure good patient selection and maximize the survival benefit.

In this review, we present and summarize the breakthroughs and challenges that mark the development and use of neoadjuvant chemotherapy, immunotherapy and targeted therapy in NSCLC. Topics relevant to their advantages and drawback, traditional and surrogate markers of treatment response, and the need to accurately monitor, predict, or prevent adverse events. By doing so we aim to spur further study that may lead to wider and better application these therapies in NSCLC patients.

### 3.4 History and current state of neoadjuvant chemotherapy in NSCLC

In the 1990s, complete surgical resection was the mainstay treatment of choice for early-stage NSCLC. A meta-analysis of 52 clinical trials conducted in 1995 suggested that supplementing resection with platinum-based chemotherapy could lead to an absolute survival benefit of 5% at 5 years compared to resection alone, but the result was not statistically significant (p = 0.08) (53). Encouraged nevertheless by the result, many researchers started conducting larger clinical trials testing various adjuvant chemotherapeutic regimens (54–58). Simultaneous enthusiasm was generated by the positive outcomes of neoadjuvant chemotherapy in head and neck cancer, and researchers started exploring the feasibility of this strategy in resectable NSCLC (10).

In an initial report published in 1989 by Faber et al., preoperative chemotherapy was shown to be feasible with acceptable toxicity and operative mortality (59). Following this report, many phase III trials were conducted to investigate various preoperative systemic therapeutic modalities. The complete resection rate of patients undergoing preoperative chemotherapy was comparable to those planned for surgery first, showing that neoadjuvant chemotherapy was well tolerated, paving the way for more trials (**Table 1**). In the first randomized controlled trials (RCT) exploring neoadjuvant therapy in NSCLC, Roth et al., and Rosell et al., found that preoperative therapy followed by surgery increased median OS when compared to surgery alone (60, 66). A French RCT for early-stage NSCLC found that patients undergoing neoadjuvant chemotherapy had a statistically significant OS benefit at 5 years (49% vs 34%, p =0.02) in N0 and N1 disease compared to those who did not receive preoperative chemotherapy (64). In the Southwest Oncology Group Trial S9900, OS (Hazard Ratio (HR) = 0.79, p = 0.11) and progression free survival (PFS) (HR = 0.80, p = 0.10) were both higher with preoperative chemotherapy (40). Surgical morbidity and mortality rates after preoperative chemotherapy were similar to rates observed after surgery alone; 5% in the neoadjuvant group and 3% in the surgery only group. Unlike the French trial, the S9900 trial did not find a difference in treatment effect by stage or node status. The Chemotherapy in Early stages NSCLC Trial (ChEST) used a primary endpoint of PFS instead of OS (65). The 3-year PFS rates were 48% (95% CI: 38.9% to 56.4%) for surgery alone versus 53% (95% CI: 43.6% to 61.3%) for preoperative chemotherapy and surgery (HR = 0.7, p = 0.003). The greatest survival benefit was seen in stage IIB/IIIA NSCLC. Perioperative toxicity from chemotherapy was responsible for a non-statistically significant excess mortality in the chemotherapy group.

**Table 1:** Details of select completed phase III neoadjuvant chemotherapy trials in resectable NSCLC.

Study	Cancer Stage	Treatment Arm	Number of Patients	R0 Resection Rate %	5-Year OS %
Rosell et al. (60)	IIIA	MIP	30	77	17
		Surgery only	30	90	0
Roth et al. (61)	IIIA	CEP	28	39	36
		Surgery only	32	31	15
Depierre et al. (62)	IB, II, IIIA	MIP	179	92	41
		Surgery only	176	86	32
Felip et al. (NATCH) (63)	IB, II, IIIA	Neoadjuvant PacCb	199	87	47
		Surgery only	210	90	44
		Adjuvant PacCb	210	90	46
Pisters et al. (S9900) (64)	IB, II, IIIA	PacCb	169	84	50
		Surgery only	167	87	41
Scagliotti et al. (ChEST) (65)	IB, II, IIIA	GP	129	NR	67 - 3-year PFS
		Surgery only	141	NR	60 - 3-year PFS

OS, Overall Survival; PFS, Progression Free Survival; CEP, cyclophosphamide, etoposide, and cisplatin; MIP, mitomycin, ifosfamide, cisplatin; PacCb, paclitaxel, carboplatin; GP, gemcitabine + cisplatin; NR, Not Reported.

The reported survival benefit of neoadjuvant chemotherapy in the above trials was similar to that of adjuvant chemotherapy (9). A major difference between preoperative and postoperative chemotherapy is compliance with the treatment regimen. In the three-arm Neoadjuvant/Adjuvant Taxol/Carboplatin Hope (NATCH) trial, 90% of patients completed the full systemic therapy regimen in the preoperative chemotherapy arm compared to 66% of patients in the postoperative arm (44). Whether or not this plays a role in OS remains a question. Unfortunately phase III trials designed to answer this failed to accrue enough sample size. In fact, both the ChEST and the S9900 trials were closed prematurely before reaching their endpoints.

The primary reason behind these closures was the publication of results from phase III trials of adjuvant chemotherapy (64, 65, 67). In a pooled analysis of 5 trials and 4584 patients by the Lung Adjuvant Cisplatin Evaluation (LACE) group, a clear OS benefit of 5.4% at five years was found with adjuvant chemotherapy compared to surgery-only (HR = 0.89, p = 0.005) (9). Like the ChEST trial, survival benefit varied by stage, with the greatest benefit seen in patients with stage II and stage IIIa disease. This led to FDA approval of adjuvant cisplatin-based regimens for stage II and stage III disease (68). A subsequent Cochrane meta-analysis of 8447 patients also definitively demonstrated a clear benefit of adding chemotherapy after surgery with an OS increase of 4% at 5 years (HR = 0.86, p < 0.0001) (69). A year after the results of the LACE analysis were published, Lim et al. conducted a pooled analysis of 32 RCTS, comparing neoadjuvant chemotherapy to adjuvant chemotherapy. Using indirect comparison meta-analysis, Lim et al. found that the relative OS hazards of postoperative compared with preoperative chemotherapy administration was 0.99 (95% CI: 0.81-1.21, p = 0.91) (33). Findings were similar for DFS, and the authors concluded that there was no evidence of a survival difference between neoadjuvant and adjuvant chemotherapy. With the clear benefit of adjuvant chemotherapy established and no additional benefit of neoadjuvant therapy, interest in neoadjuvant chemo-monotherapy dwindled.

The current state of neoadjuvant chemotherapy in NSCLC can characterized as one of limbo, stemming from a lack of initial widespread adoption as well as fears of delaying time to definitive curative resection. Presently, the standard of care for early-stage NSCLC is complete surgical resection followed by adjuvant chemotherapy for patients with stage II and stage III disease (70). The National Comprehensive Cancer Network (NCCN) guidelines considers neoadjuvant chemotherapy to be a valid alternative to patients with resectable NSCLC who are likely to receive adjuvant chemotherapy (71). Despite this and evidence showing that the neoadjuvant route is non-inferior, most physicians tend to plan for postoperative chemotherapy to minimize delay to resection (70, 72, 73). The recommendation for neoadjuvant chemotherapy also varies dramatically depending on the preferences of the treating clinician. Presently, patients with node-positive disease and comorbidities that delay surgical resection are more likely to receive preoperative chemotherapy (25, 71). The addition of platinum doublet therapy to NSCLC treatment provided a modest survival benefit whether in the adjuvant or neoadjuvant setting. Looking to expand this survival benefit in resectable NSCLC, research into immunotherapy and targeted therapy is being conducted at an accelerated pace using the neoadjuvant platform.

### 3.5 Neoadjuvant immunotherapy in NSCLC

Conventional platinum-based adjuvant chemotherapy only improves 5-year OS in early-stage NSCLC by approximately 5% (17). The aggressive biology of lung cancer coupled with its genetic heterogeneity limits the survival benefit of such therapies (74). Thus, researchers have turned to other modalities to extend survival times. While multiple immunotherapies including IL-2 and cancer vaccines have been explored as neoadjuvant cancer therapies, the development of immune checkpoint inhibitors (ICIs) over the last decade have dramatically altered the treatment landscape of NSCLC (75).

The primary targets of ICIs are programmed cell death protein 1 (PD-1), programmed death receptor-ligand 1 (PD-L1) and cytotoxic T-lymphocyte-associated protein 4 (CTLA-4) (76–78). Besides cognate interactions between the T cell receptor (TCR) and the antigen/MHC complex, T-cell activation requires co-stimulatory signals delivered when CD80/86 on an antigen presenting cell (APC) binds with CD28 on the T-cell surface. PD-1 is present on activated T-cells, and it binds to PD-L1 expressed on tumor cells or APCs. PD-L1 can engage PD-1 on the T-cell surface triggering an inhibitory signal cascade that dampens further activation and effector function, leading to suppressed and dysfunctional anti-tumor immune responses (79). CTLA-4 directly competes with CD28 to bind with CD80/86 and, in so doing, prevents the activation of T-cells (80). Agents disrupting the PD-1/PD-L1 axis or blocking the CTLA-4 checkpoints aim to bolster and re-activate the host immune system, enabling it to target tumor cells. These ICI therapies have shown considerable promise when used to treat advanced and, more recently, early-stage NSCLC patients as well.

ICI therapy in clinical lung cancer management first took root in advanced NSCLC. Data from the POPLAR/OAK, KEYNOTE-10, CHECKMATE-017, CHECKMAE-057 trials established the safety and efficacy of PD-1/PD-L1 checkpoint inhibitors in NSCLC (81–84). Further results from the KEYNOTE-024 and the KEYNOTE-189 trials confirmed that adjuvant immunotherapy in conjunction with chemotherapy doubled survival times when compared to chemotherapy alone (85, 86). Inspired by the success of adjuvant ICI therapy in advanced NSCLC, researchers turned to neoadjuvant ICI therapy in resectable NSCLC to investigate various agents.

The rationale for preoperative ICI therapy and the potential advantages in NSCLC and other cancers are many-fold (reviewed in depth in reference # (87)). Application of immunotherapy prior to other interventions creates the opportunity to generate important biospecimens (serum, peripheral blood leukocytes, tumor biopsies, etc.), pre- and post-treatment. Such samples when analyzed with the powerful research tools (next-generation sequencing, high-parameter cytometry, metabolomics, etc.), can yield insights into the determinants of therapy response or resistance, as well as helpful biomarkers predicting benefit and adverse effects. As such neoadjuvant immunotherapy can be veritable boon for investigators aiming to better understand and apply promising agents like ICIs.

There is also the notion that the intact tumor provides a source for antigen-specific T-lymphocyte immunity. In contrast to their use in the adjuvant setting, wherein the primary tumor is removed, neoadjuvant ICIs are expected to act in the context of a large antigenic load more likely to support the generation of tumor-specific T-cells and better anti-tumor immune responses (6). Liu et al., demonstrated this effect in a murine model of triple-negative breast cancer (88). Mice treated with neoadjuvant anti-PD-1 survived 40% longer than mice in the postoperative group. Neoadjuvant immunotherapy was also found to increase the number of tumor specific CD8+ T cells in peripheral blood and organs, implying a more robust immune response when the tumor is intact. Two additional pre-clinical studies confirmed that neoadjuvant therapy reduced the risk of disease relapse when compared to adjuvant therapy (89, 90).

Forde et al., published one of the first phase II studies designed to test the feasibility and safety of neoadjuvant nivolumab administration (19). Twenty-one patients were enrolled and received nivolumab preoperatively. The side effect profile was acceptable, with treatment-related adverse events of any grade occurring in only five out of twenty-two patients. There were also no delays to surgery, with twenty out of twenty-one patients able to undergo complete resection. Wislez et al., conducted another single agent phase II trial (IONESCO) of durvalumab (91). Out of forty-six patients, 89% had complete resections and no one had any grade 3-5 serious adverse events. Nineteen percent of the patients had a major pathological response and all of them were disease free at 12 months (compared to only 11% without MPR). As the neoadjuvant approach facilitates swift and cost-effective exploration, numerous phase II trials evaluating diverse PD-1/PD-L1 inhibitors are underway. **Table 2** presents findings from five such trials, while ongoing investigations involve tislelizumab (NCT03745222), SHR‐1316 (NCT04316364), camrelizumab (NCT04541251, NCT04338620), toripalimab (NCT04304248, NCT04158440), and cemiplimab (NCT03916627) (14).

**Table 2:** Details of select completed single-agent immunotherapy trials.

Study	Phase	Cancer Stage	Neoadjuvant Therapy	Number of Patients	R0 Resection Rate	ORR	MPR	pCR	Grade 3 or above TRAEs
Gao et al. (92)	I	IA-IIIB	Sintilimab	40	90%	20%	40.50%	16.20%	10%
Forde et al. (93)	II	IA-IIIA	Nivolumab	21	95.20%	9.50%	45%	10%	4·5%
Chaft et al. (LCMC-3) (94)	II	IB-IIIB	Atezolizumab	181	82·3%	6·9%	20·4%	6·8%	16·6%
Besse et al. (PRINCEPS) (95)	II	IA –IIIA	Atezolizumab	30	96·7%	0%	0%	0%	0%
Wislez et al. (IONESCO) (91)	II	IB –IIIA	Durvalumab	46	89·1%	8·7%	19%	7%	0%

ORR, Objective Response Rate; MPR, Major Pathological Response; pCR, Complete Pathological Response; TRAE, Treatment Related Adverse Event.

Despite the optimism and encouraging results seen in recent years, some obstacles must be overcome for the optimal deployment of neoadjuvant ICI in NSCLC. Establishing clinical benefit remains elusive, as many trials lack sufficient follow-up for confirming survival advantage. Rosner et al., recently reported a five-year follow-up on neoadjuvant anti-PD-1 therapy in NSCLC, indicating that MPR and pre-treatment PD-L1 trended towards improved recurrence-free survival (RFS) (80). Additional follow up results from completed phase II trials are eagerly awaited to confirm the survival benefit of various immune checkpoint inhibitors. A major factor limiting adoption of neoadjuvant ICI therapy is the fact that a large proportion of patients either fail to respond to initial therapy or develop treatment resistance (96). For this reason, the NCCN does not recommend the use of neoadjuvant immune-monotherapy in resectable NSCLC, but recommends immune checkpoint combination therapies (71).

### 3.6 Neoadjuvant ICI combination therapy in NSCLC

Response to treatment with single-agent neoadjuvant immunotherapy varies widely, with MPR ranging from 0 – 45% (**Table 2**). Many patients either fail to respond to initial treatment or develop resistance to ICI therapy. Cancer cells can alter processes related to immune recognition, cell signaling, gene expression, and T-cell activation, leading to evasion of both innate and acquired immunity (97). To overcome treatment resistance, numerous ICI combination therapies are being investigated.

As systemic chemotherapy is already the standard of treatment in early-stage NSCLC along with complete resection, researchers first investigated the combination of neoadjuvant chemotherapy and immunotherapy. The rationale for this is two-pronged. Clinically, combining chemotherapy with immunotherapy has led to better survival outcomes in stage IV NSCLC (98). Biologically, it is observed that chemotherapy acts synergistically with immunotherapy to reinforce the antitumor response (17). Chemotherapy is known to have immunostimulatory effects via increased expression of antigens in the immune tumor microenvironment, increased T-cell infiltration, and inhibition of effector cells (23). The hypothesized cellular mechanisms behind the immunostimulatory effect are blockade of signal transducer and activator of transcription 6 signaling, downregulation of PD-L1, upregulation of mannose-6-phosphate receptor expression, and activation of high-mobility group protein box-1 (24).

One of the first phase II clinical trials testing chemoimmunotherapy was the multicenter NADIM study (99). It combined neoadjuvant carboplatin/paclitaxel with three cycles of preoperative nivolumab followed by adjuvant nivolumab for 1 year in patients with stage IIIa NSCLC. Eighty-nine percent of patients achieved complete tumor resection and 30% of patients had treatment-related adverse events of grade 3 or worse. However, none of the adverse events were associated with surgery delays or deaths. Additionally, 81% of patients achieved a major pathological response. Details of other phase II chemoimmunotherapy trials are given in **Table 3**.

**Table 3:** Details of select completed chemoimmunotherapy and dual immunotherapy trials.

Study	Phase	Cancer Stage	Neoadjuvant Therapy	Number of Patients	R0 Resection Rate	ORR	MPR	pCR	Grade 3 or above TRAEs
Rothschild et al. (SAKK 16/14) (100)	II	IIIA–N2	Cisplatin/docetaxel + durvalumab	68	93.00%	58.20%	61·8%	18·2%	4·5%
Shu et al. (101)	II	IB–IIIA	Atezolizumab + carboplatin/paclitaxel	30	86·7%	63·3%	56·7%	33·3%	60%
Provencio et al. (NADIM) (99)	II	IIIA	Nivolumab + carboplatin/paclitaxel	46	89·1%	76·1%	82·9%	63·4%	30·4%
Forde et al. (CHECKMATE-816) (93)	III	IB-IIIA	Nivolumab + carboplatin/paclitaxel	179	83.20%	NR	36.90%	24%	33.50%
			Carboplatin/paclitaxel	179	77.80%	NR	8.90%	2.20%	36.9
Cascone et al. (NEOSTAR) (102)	II	I-IIIA	Nivolumab + Ipilimumab	21	81·0%	19%	38.10%	28.60%	4·8%
			Nivolumab	23	95·7%	21.70%	21.70%	8.70%	4·3%

ORR, Objective Response Rate; MPR, Major Pathological Response; pCR, Complete Pathological Response; TRAE, Treatment Related Adverse Event.

Positive results from phase II trials encouraged researchers to pursue phase III trials. In the CHECKMATE-816 trial conducted by Forde et al., neoadjuvant nivolumab plus platinum-based chemotherapy was compared to neoadjuvant chemotherapy alone (93). Patients in the chemoimmunotherapy group had an event-free survival of 36 months vs 20.8 months in the chemotherapy only group. The percentage of patients with a pCR was 24.0% (95% CI: 18.0 - 31.0) and 2.2% (95% CI: 0.6 - 5.6) respectively (odds ratio, 13.94; P<0.001). Complete resection rate and the number of grade 3 or 4 treatment related adverse events were comparable in both groups. This established the fact that chemoimmunotherapy results in better treatment response without increasing the number of treatment related complications or impeding the feasibility of surgery. These results led to the FDA approval of neoadjuvant nivolumab plus platinum-based chemotherapy use in patients with stage IB-IIIA NSCLC. Other phase III trials of various immunotherapy combinations are ongoing (**Table 4**).

**Table 4:** Details of ongoing immune checkpoint inhibitor combination therapy trials.

Trial Name	Trial ID	Phase	Cancer Stage	Neoadjuvant therapy	Adjuvant therapy	Number of Patients	Primary Endpoint
AEGEAN	NCT03800134	III	IIA–IIIB	Durvalumab + platinum doublet	Durvalumab x 1 year	800	MPR and EFS
IMPOWER-030	NCT02486718	III	IIA–IIIB (T3N2)	Atezolizumab + platinum doublet	Atezolizumab x 1 year	450	MPR and EFS
CHECKMATE-77T	NCT04025879	III	IIA–IIIB (T3N2)	Nivolumab + platinum doublet	Nivolumab x 1 year	452	EFS
KEYNOTE-671	NCT03425643	III	IIA–IIIB (T3N2)	Pembrolizumab + platinum doublet	Pembrolizumab x 39 weeks	786	EFS and OS
INCREASE	EudraCT number: 2019-003454-83	II	cT3-4N0-1M0 resectable	Ipilimumab + nivolumab followed by nivolumab + platinum doublet	None	29	Safety and pCR
INNWOP1	NCT04875585	II	IA3-IIIA	Pembrolizumab/Levatinib	Pembrolizumab x 15 cycles	33	MPR
CANOPY-N	NCT03968419	II	IB–IIIA	Canakinumab	None	110	MPR
				Canakinumab plus Pembrolizumab	None		
				Pembrolizumab	None		
NeoCOAST 2	NCT05061550	II	II to IIIB	Oleclumab + durvalumab + platinum doublet chemotherapy	Oleclumab + durvalumab	210	Safety and pCR
				Monalizumab + durvalumab + platinum doublet	Monalizumab + durvalumab		
				MEDI5752 + platinum doublet	MEDI5752		

MPR, Major Pathological Response; EFS, Event Free Survival; OS, Overall Survival; pCR, Complete Pathological Response.

Combining two distinct immune checkpoint inhibitors is another way to avoid treatment resistance and increase efficacy. Cascone et al., designed a preclinical study to examine dual vs. single agent and neoadjuvant vs adjuvant immunotherapy (103). Mice that received a neoadjuvant dual immunotherapy regimen survived longer than those on adjuvant immunotherapy or single agent neoadjuvant immunotherapy. The combination of an anti-PD-1/PD-L1 agent with an anti-CTLA-4 agent is the most investigated out of all potential dual ICI regimens. PD-1 and CTLA-4 both inhibit T-cell activation, yet function through distinct, complementary mechanisms (104). Anti-CTLA-4 agents cause activation and proliferation of T-cells whereas anti-PD-1 agents are thought to aid in the recognition and elimination of tumor cells. Therefore, a combination of both makes logical sense.

Given the established safety of nivolumab monotherapy and the survival benefit of dual immunotherapy demonstrated in metastatic melanoma, Hellmann et al., designed the CHECKMATE-012 trial for patients with advanced NSCLC. This phase one trial assessed the safety of combining nivolumab (anti-PD-1) with ipilimumab (anti-CTLA-4) and found that the combination had a tolerable safety profile (grade 3 and above treatment related adverse events occurred in 27/78 patients) (105). OS benefit of combination nivolumab plus ipilimumab was confirmed in results from a published phase III trial (nivolumab + ipilimumab vs. chemotherapy OS (months); 17.1 vs. 14.9, p = 0.007) (106). Strikingly, OS benefit was seen in patients independent of PD-L1 expression level, implying a more robust treatment response. Using these results as inspiration, researchers started investigating combination anti-PD-1/anti-CTLA-4 therapy in resectable lung cancer.

NEOSTAR was the first phase 2 RCT comparing neoadjuvant nivolumab alone to neoadjuvant nivolumab plus ipilimumab in resectable lung cancer. In the nivolumab plus ipilimumab arm, thirty-eight% (8/21) of the patients achieved MPR whereas in the nivolumab arm, 22% achieved MPR (5/23). Though the sample size was small, the results for dual ICI immunotherapy are encouraging. In a 2022 meta-analysis of 16 neoadjuvant systemic therapy clinical trials, pooled pCR rates for ICI plus chemotherapy, mono-ICI, dual ICIs, and chemotherapy alone were 28.6% (95% CI: 20.0–38.7%), 9.9% (95% CI: 5.7–15.3%), 28.6% (95% CI: 13.8–50.7%), and 2.0% (95% CI: 1.0–5.7%), respectively. These results emphasize the superiority of combination ICI therapy over other treatment modalities (4).

To overcome ICI treatment resistance, researchers are investigating the combination of ICIs with other agents as well. Due to the multitude of drug combinations possible to test, platform style trial designs are becoming popular in which patients receive neoadjuvant ICI and are subsequently randomized to a trial arm with a novel therapeutic agent. For example, in the phase II platform trial, NEOSTAR, the combination of durvalumab with oleclumab (anti-CD73), monalizumab (NKG2A inhibitor) and danvatirsen (anti-STAT3) were tested simultaneously (107). Higher MPR rates were found in all combination therapy arms vs. durvalumab alone, suggesting that combination strategies boost PD-1/PD-L1 inhibitor neoadjuvant efficacy. Nivolumab is also being explored in combination with BMS‐813160 (CC chemokine receptor2/5‐inhibitor) or BMS‐986253 (anti‐interleukin‐8) in the neoadjuvant setting (NCT04123379) (17).

Anti-angiogenic therapy in combination with immunotherapy has also been shown to be effective in advanced NSCLC (108). This has motivated researchers to investigate a combination of ICIs and anti-angiogenic agents in resectable NSCLC. Indeed, a couple of ongoing trials aim to test the promise of combined apatinib and camrelizumab treatment (NCT04506242) and sintilimab plus bevacizumab and platinum doublet therapy (NCT03872661).

Based on these findings, it can be said that there is more clinical enthusiasm for neoadjuvant ICI combination therapy than immune monotherapy. Neoadjuvant nivolumab is approved by the US Food and Drug Administration for use in combination with platinum-based chemotherapy for resectable NSCLCs (46). In the 2023 NCCN update for resectable NSCLC, a recommendation was added that all patients with node-positive disease or tumors >4cm should be evaluated for preoperative nivolumab therapy on the basis of the results of the phase II NADIM and preliminary results of the phase III Checkmate-816 trial (71). Data for node-negative or lower T stage disease is less conclusive, and neoadjuvant ICI combination therapy is not currently recommended. Most published clinical trials investigating neoadjuvant ICI combination therapy are small, single arm safety and efficacy studies. Follow-up survival data of many ongoing phase III trials (**Table 4**) is eagerly awaited to guide future treatment protocols. As combination therapies have proven to be relatively tolerable and more effective than monotherapy, they may be essential in future neoadjuvant-based treatments of resectable NSCLC.

### 3.7 Immune-related adverse events

One area of concern with both single agent and combination ICI therapies is potential toxicity. Due to the disruption of key immunoregulatory circuitry, ICIs can trigger autoimmune toxicities called immune-related adverse events (irAE) in any tissue (109). Most irAEs are manageable with steroids if caught early, however, some endocrine disorders are irreversible (requiring lifelong hormone replacement). In a pooled analysis of 16 lung cancer trials and 6226 subjects, the incidence of any grade irAE and severe irAE was 37.1% and 18.5% respectively (110). The incidence of adverse events was significantly higher in patients on chemoimmunotherapy than patients on only immunotherapy, especially monotherapy (110). Since the success of surgical intervention can be time- and disease stage-sensitive, even short duration irAEs in the neoadjuvant setting can be harmful due to an increase in surgical delay. Thus, careful monitoring for symptoms of irAEs is critical. Additional research into the underlying causes of irAEs may even allow for the prevention of such complications as well.

It is important to note that increased irAE incidence, however, does not necessarily translate into decreased survival. A meta-analysis conducted by Zhao et al., found that patients who developed irAEs after immune-checkpoint inhibition had significantly improved OS (HR: 0.51; 95% CI: 0.44-0.60; P < 0.01) and PFS HR: 0.50; 95% CI: 0.43-0.58; P < 0.01) compared to those who did not (111). In another meta-analysis by Jiang et al., though the incidence of treatment related adverse events was higher in chemoimmunotherapy combinations relative to immunotherapy (73.9% vs. 42.9%), the incidence of severe adverse events only increased by a small amount (18% vs. 12.3%). The incidence of severe adverse events was also similar between monotherapy with ICI inhibitors and dual immunotherapy (12.3% vs. 9.9%) (4). From the above data, we can draw the conclusion that immune related adverse events should not preclude patients from receiving neoadjuvant immunotherapy as the adverse events in question are not likely to be too severe, and they may potentially indicate that a treatment is working.

### 3.8 Biomarkers for neoadjuvant ICI therapy in NSCLC

The current data presents a basis for optimism in the realm of neoadjuvant ICI therapy. However, responses to these therapies exhibit notable heterogeneity, and the potential for immune-related adverse events underscores the urgency in identifying biomarkers for neoadjuvant ICI response, aiming to optimize clinical benefits (26, 112). In this context, two primary biomarkers come to the forefront: PD-L1 status and tumor mutational burden (TMB), typically assessed through tumor tissue biopsies.

Given the central role of the PD-1/PD-L1 axis in ICI mechanisms, PD-L1 status as a predictive biomarker has garnered considerable attention. Initial findings from pivotal trials yielded conflicting results and have necessitated validation of PD-L1 status in larger cohorts. For instance, the landmark trial by Forde et al., showed no association between PD-L1 percent and tumor regression (19). These findings were later corroborated in phase II trials by Shu et al., and Lee et al. (101, 113),. Yet, other phase I and phase II trials showed the opposite result. In a phase I trial, Gao et al., showed that PD-L1 expression in stromal cells at the primary site at baseline was correlated with the percentage of pathologic response of the primary lesion (Pearson correlation = –0.37, p = 0.05) (92). Similarly, Cascone et al., found that PD-L1 expression is higher in pathological responders compared to non-responders (114). To resolve this, a pooled analysis of phase I, II and III trials was conducted by a group led by Passiglia in 2016 (115). In this analysis of seven trials with 914 patients, patients with PD-L1 positive tumors (PD-L1 tumor cell staining ≥1%), had a significantly higher ORR compared to patients with PD-L1 negative tumors (odds ratio 2.44, 95% CI: 1.61-3.68). The latest phase III trial published by Forde et al., in 2022 further consolidates the significance of PD-L1 as a predictive biomarker for ICI therapy response. A benefit with nivolumab plus chemotherapy was seen across PD-L1 subgroups, with a greater event-free survival benefit in patients with a tumor PD-L1 expression level of 1% or more than in those with a level of less than 1% (93).

Another marker of interest is TMB, which reflects the number of mutations in the cancer cell genome. TMB quantifies the number of mutations per megabase (Mut/Mb) of sequenced tumor DNA. With a higher number of mutations detected, there is consequentially an increased generation of immunogenic neo-antigens (26). In advanced NSCLC, there is evidence to show an OS benefit of ICI agents over chemotherapy alone in patients with high TMB (116). Data in the neoadjuvant setting is still preliminary and conflicting. Forde et al., found that patients treated with ICI therapy with MPR had a significantly higher TMB than patients without MPR (19). Gao et al., found that high TMB was associated with better event free survival in patients treated with neoadjuvant sintilimab (92). Other authors such as Provencio et al., and Chaft et al., did not find a significant association between TMB and pathological response or survival (94, 99). This variability in results may be attributed to the small-scale nature of these studies and limited cohorts, highlighting the necessity for larger prospective studies to validate the role of TMB in this context.

Beyond these more established biomarkers, there is a burgeoning interest in exploring additional markers of ICI efficacy that may be relevant to the neoadjuvant setting. Indeed, many recent studies have revealed a number of apparent candidates including but not limited to the size of the T cell receptor repertoire, the presence of tumor-infiltrating lymphocytes and expression of T cell effector factors, tumor neoantigen burden, circulating tumor DNA (ctDNA), neutrophil-lymphocyte ratio, and favorable gut microbiome species (a detailed review can be found (26). Additional sources of potential biomarkers telling predictive of ICI outcomes may also emerge from ongoing study of extracellular vesicles (exosomes and microvesicles) and microRNA signatures. Despite considerable interest, data on these marker and marker sources, and their relevance to neoadjuvant ICI specifically remains limited or incomplete. With further study, however, may bring them to the forefront of the clinical setting.

### 3.9 Neoadjuvant targeted therapy in NSCLC

The advent of molecular testing revolutionized the treatment of advanced lung cancer (117). All patients with advanced NSCLC undergo testing for the presence of driver mutations such as epidermal growth factor receptor (EGFR), anaplastic lymphoma kinase (ALK), and ROS proto-oncogene 1 (ROS1). NSCLC patients with sensitizing EGFR mutations are treated with EGFR tyrosine kinase inhibitor (EGFR-TKI) therapy. First and second-generation EGFR-TKIs such as gefitinib, erlotinib, afatinib and icotinib have been proven to extend PFS when compared to chemotherapy (118–122). Results of the FLAURA and ADAURA trials let to the approval of adjuvant osimertinib for patients with positive EGFR mutations (123, 124). With the survival benefit of targeted therapy being proven in advanced NSCLC, most clinical trials for neoadjuvant therapy in early-stage lung cancer screen for actionable mutations as an exclusion criterion. These patients are instead offered surgery followed by adjuvant targeted therapy. Neoadjuvant targeted therapy for early-stage NSCLC is still being explored and has not yet entered mainstream clinical practice. Preliminary data from several phase II clinical trials, however, are cautiously optimistic and may pave the way forward for neoadjuvant therapy targeting driver oncogenes. Lara-Guerra et al., published one of the first phase II studies of preoperative gefitinib in stage I NSCLC (125). He found that the safety profile was acceptable, with only three out of thirty-six patients developing grade 3 toxicity and above. The EMERGING-CTONG 1103 study was a randomized phase II study designed to assess the efficacy of neoadjuvant/adjuvant EGFR-TKI therapy in relation to neoadjuvant/adjuvant chemotherapy (126). Complete resection rate in the erlotinib arm was 73%, compared with 63% in the chemotherapy arm. Median PFS was significantly longer in the erlotinib arm (21.5 months, 95% CI = 16.7 - 26.3) compared to the chemotherapy arm (11.4 months, 95% CI = 7.3 - 15.5 months; hazard ratio [HR], 0.39; 95% CI, 0.23 to 0.67; P <.001). Erlotinib was also found to be well tolerated with zero patients experiencing an adverse event grade 3 or higher whereas 29.4% of patients treated with chemotherapy did.

Sun et al., performed a pooled analysis of five phase II trials testing neoadjuvant targeted therapy involving 124 patients with resectable NSCLC (124). The pooled ORR was 58.5% [95% CI = 45.5% 71.8%] and the complete resection (R0) rate was 64.3% (95% CI = 43.8%-84.8%), respectively. The pooled median PFS and OS were 13.2 and 41.9 months, respectively. Neoadjuvant targeted therapy was well tolerated by patients and the incidence of grade three-fourths adverse events was 5.3% for hepatotoxicity and 14.7% for skin rash. Surgery was not delayed for any patient due to treatment-related adverse events. While numerous phase II trials appear to show that neoadjuvant targeted therapy is feasible, data from phase III randomized controlled trials are needed to definitively establish the survival benefit from these therapies. One such trial, the NEOADAURA trial is an ongoing RCT investigating neoadjuvant chemotherapy alone, neoadjuvant chemotherapy plus osimertinib and osimertinib alone (127). The primary outcome measure is MPR, and the secondary outcome measures are pCR, DFS and OS.

Another set of driver mutations are anaplastic lymphoma kinase (ALK) gene rearrangements. ALK gene rearrangements are associated with younger age, non-smoking status, and worse prognosis in NSCLC patients (128). In advanced NSCLC, ALK-positive NSCLC patients are treated with adjuvant first-generation crizotinib or the newer second-generation alectinib (129). A few case reports and retrospective studies have demonstrated the activity of neoadjuvant alectinib and crizotinib, but no phase two trials have been conducted (130–132). The ongoing alectinib in neo-adjuvant treatment of stage III NSCLC (ALNEO) trial is a phase II trial designed to assess the efficacy and safety of preoperative administration of alectinib (**Table 5**).

**Table 5:** Details of ongoing targeted therapy trials.

Oncogene	Trial Name	Trial ID	Phase	Cancer Stage	Neoadjuvant therapy	Adjuvant therapy	Number of Patients	Primary Endpoint
**EGFR mutation**	ANSWER	NCT04455594	II	IIIA (N2)	Almonertinib	None	168	ORR
					Erlotinib + platinum doublet	None		
	NEOLPOWER	NCT05104788	II	II - IIIB (N2)	Icotinib + platinum doublet	None	27	MPR
		NCT04201756	II	Resectable Stage III	Afatinib	None	47	ORR
		NCT03749213	II	IIIA (N2)	Icotinib	None	36	ORR
	PROGRESS	NCT02804776	II	Resectable NSCLC	Gefitinib	None	15	EGFR TKI sensitivity biomarkers determination
	NEOADAURA	NCT04351555	III	II–IIIB (N2)	Osimertinib	Osimertinib	328	MPR
					Osimertinib + CT	Osimertinib		
					Placebo + CT	Osimertinib		
**ALK rearrangement**	ALNEO	NCT05015010	II	III	Alectinib	None	33	MPR
**MET exon fourteen mutation**	GEOMETRY-N	NCT04926831	II	IB-IIIA(N2), IIIB (T3N2, T4N2)	Capmatinib	Capmatinib	38	MPR
**Multiple mutations (ALK, ROS1, MET, BRAF, RET, NTRK)**	NAUTIKA1	NCT04302025	II	II-III	Alectinib/Entrectinib/Vemurafenib/Cobimetinib/Pralsetinib/Atezolizumab	Alectinib/Entrectinib/Vemurafenib/Cobimetinib/Pralsetinib/Atezolizumab	80	MPR

ALK, Anaplastic Lymphoma Kinase; BRAF, BRAF Proto-Oncogene; ORR, Objective Response Rate; MET, Mesenchymal Epithelial Transition; NTRK, Neurotrophic Tyrosine Receptor Kinase; MPR, Major Pathological Response; EGFR, Epidermal Growth Factor Receptor; ROS1, ROS Proto-Oncogene 1; TKI, Tyrosine Kinase Inhibitor.

Other than EGFR and ALK TKI targeted therapies, an ever growing list of novel drugs targeting KRAS, ROS1, BRAF V600E, MET, RET, and NTRK driver oncogene mutations is being compiled (133). In addition to established targets, newly recognized oncogenic drivers such as CLIP1-LTK fusion are also being investigated as potential avenues for therapy (134). However, due to the relative scarcity of these alterations compared to EGFR mutation or ALK rearrangement, data is limited in the neoadjuvant space, and conclusions are hard to draw at present. Since performing a clinical trial exclusively for one target mutation is quite difficult, umbrella trials designed to investigate multiple molecular alterations simultaneously are underway. Such trials encompass many ‘sub-studies’, all investigating the same disease with each sub-study essentially a different treatment arm in the same trial (135). One such example, the NAUTIKA1 study. The aim is to investigate neoadjuvant and adjuvant alectinib, entrectinib, vemurafenib, cobimetinib, pralsetinib, and atezolizumab in patients with stage IB-IIIA (and selected resectable IIIB cases) NSCLC with RET, BRAF V600, NTRK, ALK, and ROS1 molecular alterations (27). Here, each arm of the study investigated a different immune checkpoint inhibitor in the context of neoadjuvant vs. adjuvant therapy. Patients were assigned to a particular treatment arm based on their particular genetic mutation. The advantage of this trial design is that it allows for simultaneous evaluation of multiple molecular targets and is hypothesized to accelerate drug development. Umbrella trials are also hypothesized to increase benefit-risk ratio as patients are slotted into the arm predicted to provide them with the greatest benefit (depending on the specific molecular makeup of their cancer) (136, 137) A summary of ongoing targeted therapy clinical trials is given in **Table 5**.

**Figure 1:**
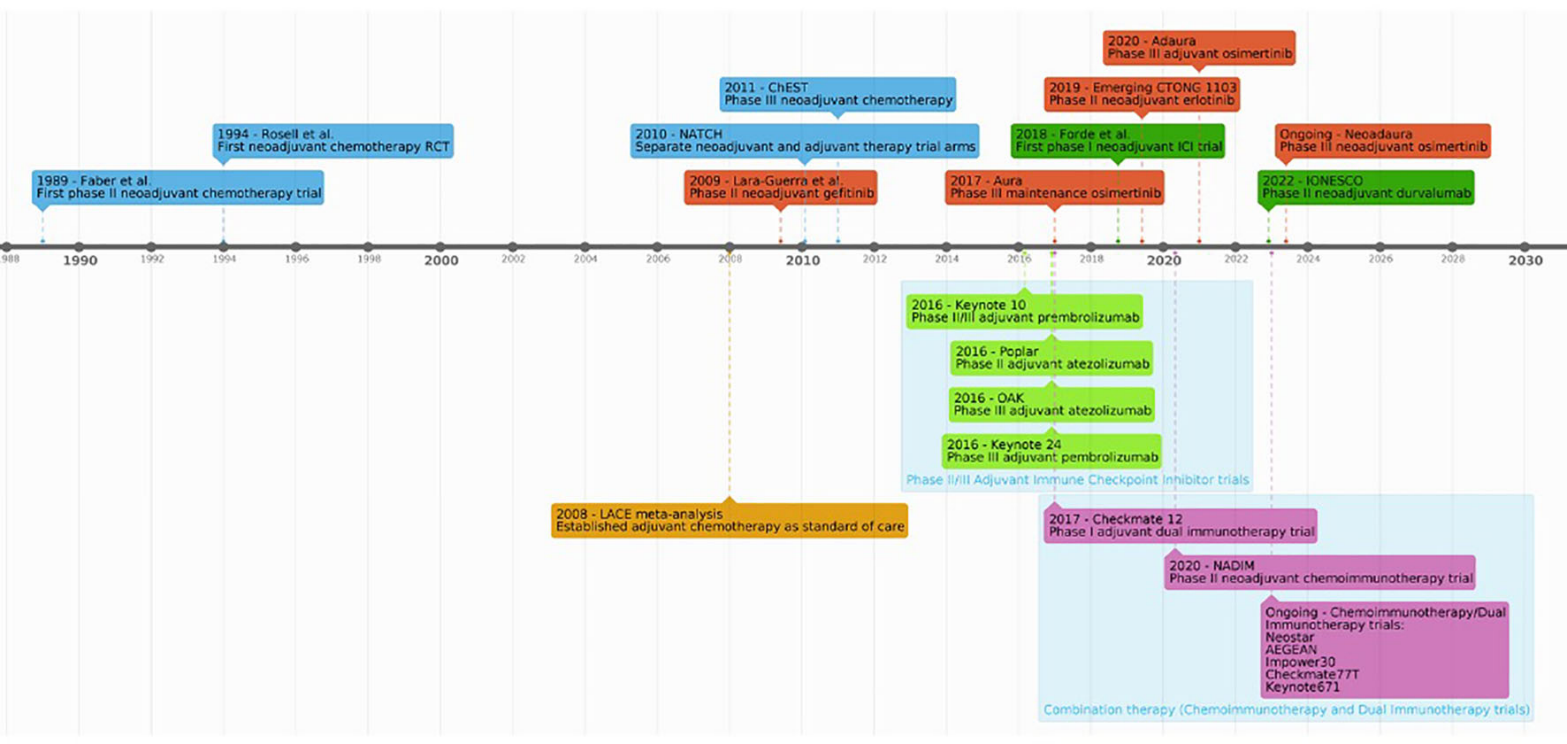
A timeline of key studies advancing the development neoadjuvant treatments for NSCLC.

As demonstrated above, similar to the developments of chemo- and immunotherapeutic strategies (summarized in **Figure 1**), use of targeted therapies to treat resectable NSCLC in the neoadjuvant setting has been marked by numerous advances and encouraging breakthroughs. Neoadjuvant targeted therapy seems to provide added survival benefit in patients with positive driver mutations. However, most of this published data is in the setting of small phase I and II safety and efficacy trials. As a result, use of neoadjuvant targeted therapy is not standard in current clinical practice. Results from phase III RCTs are awaited to establish clinical survival benefit (25).

## 4 Conclusion

Neoadjuvant therapies are showing strong promise as a treatment strategy effective for the management of resectable non-small cell lung cancer. As described above, neoadjuvant therapy offers several potential advantages over adjuvant therapy that include better targeting of micro-metastases and nodal/tumor downstaging, a more comprehensive preoperative workup facilitating lifestyle modifications, such as smoking cessation and exercise programs. In addition, the neoadjuvant setting serves to facilitate quick drug development, by enabling faster evaluation of treatment efficacy through surrogate markers of overall survival like pCR and MPR. By enabling researchers to access both pre and posttreatment tumor samples, an increasingly personalized biomarker driven approach to systemic therapy is also facilitated. Combination therapies like dual immunotherapy and chemoimmunotherapy have proven to be more effective than monotherapy and are being evaluated rapidly through umbrella trials. We eagerly await the results of multiple ongoing clinical trials incorporating diverse neoadjuvant combination therapy into their study designs. However, neoadjuvant therapy is not without its challenges, including the potential for delayed surgical resection. Ongoing research and phase III trials are crucial to further establish the survival benefits and optimize the implementation of neoadjuvant strategies, whether involving chemotherapy, immunotherapy, targeted therapy, or combinations thereof, to improve outcomes for patients with resectable NSCLC.

## Author contributions

SK: Investigation, Writing – original draft, Writing – review & editing. YV: Conceptualization, Writing – review & editing. SY: Conceptualization, Funding acquisition, Supervision, Writing – original draft, Writing – review & editing. JB: Conceptualization, Funding acquisition, Supervision, Writing – original draft, Writing – review & editing.
